# National trends in utilization and readmission following intraoperative cholangiography in gallstone pancreatitis

**DOI:** 10.1016/j.sopen.2025.05.002

**Published:** 2025-05-10

**Authors:** Ayesha P. Ng, Troy N. Coaston, Konmal Ali, Christian de Virgilio, Peyman Benharash

**Affiliations:** a Department of Surgery, David Geffen School of Medicine at UCLA, Los Angeles, CA, USA; b Department of Surgery, Harbor-UCLA Medical Center, Torrance, CA, USA

**Keywords:** Gallstone pancreatitis, Intraoperative cholangiography, Cholecystectomy, Bile duct, Readmission, Outcomes

## Abstract

**Background:**

In the absence of cholangitis, the role of intraoperative cholangiography (IOC) to exclude retained stones in mild gallstone pancreatitis (GSP) remains controversial. Using a nationally representative database, we examined the contemporary utilization of IOC and index outcomes and readmission following cholecystectomy for GSP.

**Methods:**

All adults undergoing nonelective cholecystectomy for mild GSP in the 2017–2021 Nationwide Readmissions Database were identified. Patients were stratified based on the use of IOC. Multivariable regressions and Royston-Parmar analysis were used to evaluate the association of IOC use with outcomes of interest.

**Results:**

Of 152,687 patients, 24.7 % underwent IOC. Utilization of IOC significantly decreased from 26.5 % to 20.7 % over the study period (*p* < 0.001). Compared to patients without IOC, IOC patients were older and more commonly treated at high-volume, private hospitals. Following risk adjustment, the odds of major adverse events, including mortality, complications, and bile duct injury repair were comparable between cohorts. Furthermore, length of stay and hospitalization costs were comparable between patients with and without IOC. Notably, IOC was significantly associated with 20 % decreased odds of 90-day readmission for recurrent pancreatitis or retained stone, which persisted over time (AOR 0.80 [95 % CI 0.74–0.86]).

**Conclusions:**

IOC was associated with significantly reduced readmission and comparable resource use following cholecystectomy for GSP. Despite its decreasing utilization, IOC may be a cost-effective strategy to help reduce risk for recurrent biliary disease among patients with mild GSP.

## Highlights

• Utilization of intraoperative cholangiography decreased from 2017 to 2021.
• Mortality and complications were comparable regardless of cholangiography use.
• Length of stay and total costs were similar regardless of cholangiography use.
• Intraoperative cholangiography demonstrated 20 % reduced odds of 90-day readmission.

## Graphical abstract

**Figure:**
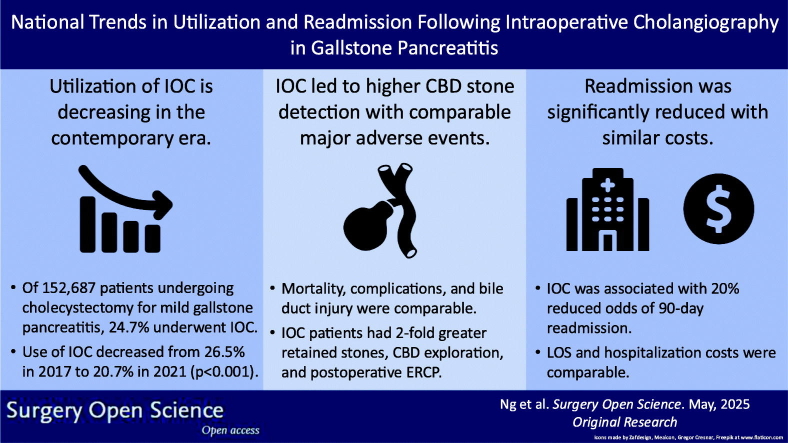

## Introduction

Gallstone pancreatitis (GSP) is the leading cause of acute pancreatitis and accounts for nearly 270,000 emergency department visits in the United States each year [1,2]. GSP is thought to be caused by small gallstones that transiently obstruct the ampulla of Vater [3]. In the absence of end-organ dysfunction, the definitive treatment for GSP is cholecystectomy within the same hospitalization, which significantly reduces the risk of recurrence [4,5]. For patients with signs of obstructive jaundice or cholangitis, clearance of common bile duct stones via preoperative endoscopic retrograde cholangiopancreatography (ERCP) is indicated [6]. In the setting of mildly elevated bilirubin levels, intraoperative cholangiography (IOC) can exclude retained stones and determine whether common bile duct (CBD) exploration or postoperative ERCP is necessary [7].

However, the role of routine IOC in patients with mild GSP remains controversial [[8], [9], [10]]. Published guidelines for the management of GSP continue to support the use of IOC to identify any remaining CBD stones [11,12]. In theory, if a gallstone remained in the CBD, this could likely lead to recurrent pancreatitis or symptomatic choledocholithiasis. Nevertheless, in a prospective study of 300 patients, Johnson and Walsh demonstrated no benefit to IOC in reducing recurrent pancreatitis within four years of initial GSP presentation [13]. Yet, existing literature evaluating the use of IOC in GSP relies on limited data cohorts. Given the sporadic use of IOC across surgeons and hospitals, a contemporary analysis at the national level is necessary to characterize whether GSP requires bile duct evaluation by IOC and if failure to do so would result in greater recurrence and readmission.

Using a nationally representative cohort of patients with mild GSP undergoing cholecystectomy, the present study examined contemporary trends in the utilization of IOC across the United States. In addition, we evaluated the association of IOC use with in-hospital outcomes and readmission. We hypothesized the use of IOC to be associated with comparable mortality, complications, and length of stay and reduced 90-day readmission and overall costs.

## Methods

### Data source and study population

This was a retrospective cohort study using the 2017–2021 Nationwide Readmissions Database (NRD). Maintained by the Healthcare Cost and Utilization Project, the NRD is the largest all-payer readmissions database that provides survey-weighted estimates for approximately 60 % of all US hospitalizations [14]. Unique linkage numbers are used to track all hospital readmissions within a one-year surveillance period. Due to the de-identified nature of the NRD, this study was deemed exempt from full review by the Institutional Review Board at the University of California, Los Angeles.

**Fig. 1:**
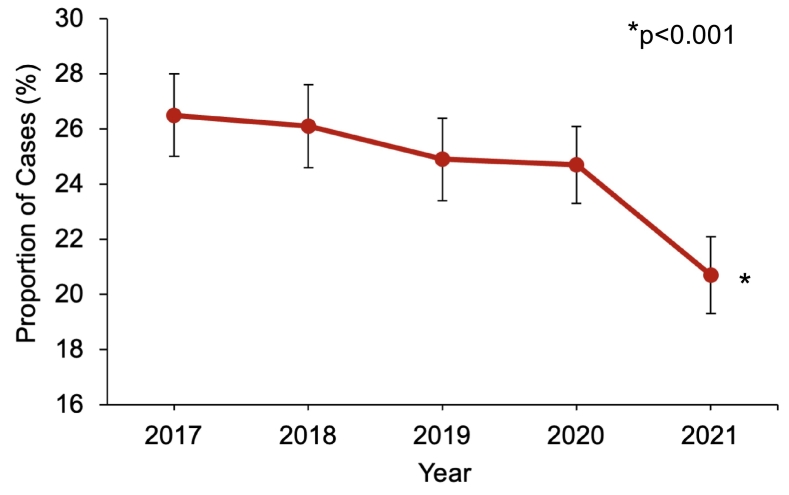
Temporal trend in utilization rate of intraoperative cholangiography during cholecystectomy for mild gallstone pancreatitis.

All nonelective adult (≥18 years) hospitalizations entailing cholecystectomy for mild GSP without necrosis or infection were identified using *International Classification of Diseases, 10th Revision (ICD-10)* diagnosis and procedure codes (Supplemental Table 1). Patients who received preoperative ERCP or with concomitant cholangitis, hepatopancreatobiliary or duodenal malignancies, or liver transplantation were excluded (Fig. 1). In accordance with the Atlanta classification of acute pancreatitis, patients with end-organ dysfunction were considered to have moderate to severe GSP and were subsequently excluded [15]. Study cohorts were then stratified based on the use of IOC (*IOC* vs *No IOC)*.

### Study variables and outcomes

Patient and hospital characteristics including age, sex, income quartile, primary payer, and hospital teaching status and ownership were defined using the NRD Data Dictionary [14]. Comorbidities such as diabetes, hypertension, coronary artery disease, congestive heart failure, and chronic lung and kidney disease were identified using ICD-10 diagnosis codes (Supplemental Table 1). The Elixhauser Comorbidity Index, a validated composite of 30 comorbidities, was used to quantify the overall burden of chronic conditions [16]. Hospitals were stratified into low-, medium-, and high-volume tertiles based on annual institutional case volume of cholecystectomy.

Treatment characteristics, including CBD exploration, indocyanine green (ICG) angiography, conversion to open, and postoperative ERCP were ascertained using relevant ICD-10 codes (Supplemental Table 1). Retained stone in addition to complications such as infectious, respiratory, cardiac, hemorrhagic, and thromboembolic were identified (Supplemental Table 1). Based on prior work, repair of bile duct injury was identified using ICD procedure codes for surgical biliary reconstruction with hepaticojejunostomy or choledochojejunostomy following cholecystectomy within the one-year NRD surveillance period [17]. Major adverse events (MAE) were defined as a composite of mortality, any complications, and repair of bile duct injury. Hospitalization costs were calculated by applying center-specific cost-to-charge ratios to overall charges and inflation-adjusted using the 2021 Personal Healthcare Price Index [18]. Indications for readmission within 90 days of discharge were tabulated using diagnosis-related groups (Supplemental Table 2). The primary outcome of interest was 90-day readmission, while MAE, conversion to open, biliary interventions, length of stay (LOS), costs, and non-home discharge were secondarily assessed.

### Statistical analysis

Categorical and continuous variables are reported as group proportions (%) or medians with interquartile range (IQR) and compared using the Pearson's chi-square or Mann-Whitney *U* tests, respectively. Significance of temporal trends was assessed using Cuzick's nonparametric test [19]. Multivariable linear and logistic regression models were developed to evaluate the association of IOC use with outcomes of interest. Variable selection was performed by applying the Least Absolute Shrinkage and Selection Operator (LASSO) regularization algorithm to reduce the risk of model overfitting and enhance out-of-sample generalizability [20]. The cumulative risk of nonelective readmission within 90 days of index discharge was evaluated using Royston-Parmar's flexible parametric regression [21]. This methodology allows for varying hazards of readmission over time and accounts for differences in patient and hospital characteristics between groups. An interaction term between the admission year and IOC use was used to analyze differences in readmission over time. Regression results are reported as adjusted odds ratios (AOR) or beta coefficients (β) with 95 % confidence intervals (95 % CI). Statistical significance was set at α = 0.05. All statistical analyses were performed using Stata 16.1 (StataCorp, College Station, TX).

## Results

### Demographic comparison

Of 152,687 patients undergoing cholecystectomy for mild gallstone pancreatitis, 37,683 (24.7 %) underwent IOC. Utilization of IOC significantly decreased from 26.5 % in 2017 to 20.7 % in 2021 (*p* < 0.001, Fig. 1). Compared to *No IOC*, *IOC* patients were older with similar burden of comorbidities (Table 1). Patients with IOC were more commonly privately insured (40.9 vs 39.3 %, *p* < 0.001) and treated at private investor-owned hospitals (15.8 vs 12.5 %, p < 0.001). In addition, *IOC* was more frequently utilized at non-metropolitan hospitals relative to *No IOC* (9.6 vs 7.7 %, p < 0.001, Table 1)*.* These findings persisted on multivariable analysis (Supplemental Table 3). Furthermore, IOC was significantly associated with treatment at high-volume hospitals relative to low-volume (AOR 1.15 [95 % CI 1.05–1.26]).

**Table 1:** Patient and hospital characteristics stratified by use of intraoperative cholangiography (IOC) during cholecystectomy for mild gallstone pancreatitis. *IQR: Interquartile range.*

Parameter	No IOC (*n* = 115,004)	IOC (*n* = 37,683)	*p*-value
Age (years, median, IQR)	53 [36–67]	54 [36–68]	<0.001
Female sex (%)	65.5	65.6	0.96
*Income Quartile (%)*			0.05
Fourth (highest)	18.7	17.6	
Third	25.5	25.5	
Second	28.0	29.0	
First (lowest)	27.7	27.8	
*Payer Status (%)*			<0.001
Private	39.3	40.9	
Medicare	31.1	31.7	
Medicaid	20.3	17.3	
Uninsured	5.8	6.2	
Other	3.4	3.9	
*Comorbidities (%)*			
Elixhauser Comorbidity Index (median, IQR)	2 [1–3]	2 [1–3]	<0.001
Diabetes	18.4	17.9	0.12
Hypertension	7.7	7.6	0.66
Coronary artery disease	8.2	8.9	0.01
Congestive heart failure	4.4	4.6	0.18
Chronic lung disease	11.8	12.1	0.25
Chronic kidney disease	1.1	1.0	0.17
*Hospital Volume Status (%)*			0.63
Low	34.3	33.6	
Medium	30.3	30.3	
High	35.5	36.1	
*Hospital Teaching Status (%)*			<0.001
Non-metropolitan	7.7	9.6	
Metropolitan non-teaching	23.1	23.3	
Metropolitan teaching	69.1	67.1	
*Hospital Ownership (%)*			<0.001
Government	10.8	9.5	
Private non-profit	76.7	74.6	
Private investor-owned	12.5	15.8	

### Unadjusted index hospitalization outcomes

Unadjusted clinical and financial outcomes are outlined in Table 2. Overall MAE rates were comparable, including similar rates of mortality, complications, and repair of bile duct injury. Compared to *No IOC*, the *IOC* cohort demonstrated higher rates of retained stones (0.5 vs 0.2 %, *p* < 0.001) and more frequently underwent common bile duct exploration (0.2 vs 0.1 %, *p* < 0.001) and postoperative ERCP (11.4 vs 4.5 %, *p* < 0.001). Of note, patients with IOC experienced significantly lower rates of conversion to open compared to *No IOC* (2.0 vs 2.9 %, p < 0.001). Among the open cases, *IOC* patients had higher rates of CBD exploration (8.2 vs 2.7 %, *p* < 0.001) and repair of CBD injury (2.8 vs 2.3 %, p < 0.001) compared to *No IOC*. While LOS was similar at approximately 4 days, the *IOC* group accrued a modest increase in hospitalization costs relative to *No IOC* ($14,300 vs $14,200, *p* < 0.001). Rates of non-home discharge were comparable between cohorts. Among the *No IOC* group*,* 2331 (2.0 %) of patients utilized ICG angiography (Table 2). Among all patients, the use of ICG significantly increased from 0.1 % in 2017 to 7.8 % in 2021 (p < 0.001).

**Table 2:** Unadjusted outcomes stratified by use of intraoperative cholangiography (IOC) among patients undergoing cholecystectomy for mild gallstone pancreatitis. *IQR: Interquartile range. LOS: Length of stay. ERCP: Endoscopic Retrograde Cholangiopancreatography. MAE: Major adverse events include mortality, any complications, and repair of bile duct injury. ICG: Indocyanine green.*

Outcome	No IOC (n = 115,004)	IOC (n = 37,683)	p-value
MAE (%)	3.6	3.7	0.60
In-hospital mortality (%)	0.1	0.1	0.58
*Complications (%)*			
Retained stone	0.2	0.5	<0.001
Infectious	0.7	0.7	0.60
Respiratory	1.5	1.4	0.24
Cardiac	0.4	0.4	0.75
Hemorrhagic	0.6	0.5	0.50
Thromboembolic	0.3	0.2	0.07
Repair of bile duct injury (%)	0.1	0.1	0.58
Common bile duct exploration (%)	0.1	0.2	<0.001
ICG angiography (%)	2.0	0.3	<0.001
Conversion to open (%)	2.9	2.0	<0.001
Postoperative ERCP (%)	4.5	11.4	<0.001
LOS (days, median, IQR)	3.8 [2.8–5.3]	3.8 [2.7–5.2]	0.04
Costs ($1000s, median, IQR)	14.2 [10.8–19.3]	14.3 [10.9–19.4]	<0.001
Non-home discharge (%)	3.1	3.1	0.90
90-day readmission (%)	8.9	7.2	<0.001

### Adjusted index hospitalization outcomes

**Fig. 2:**
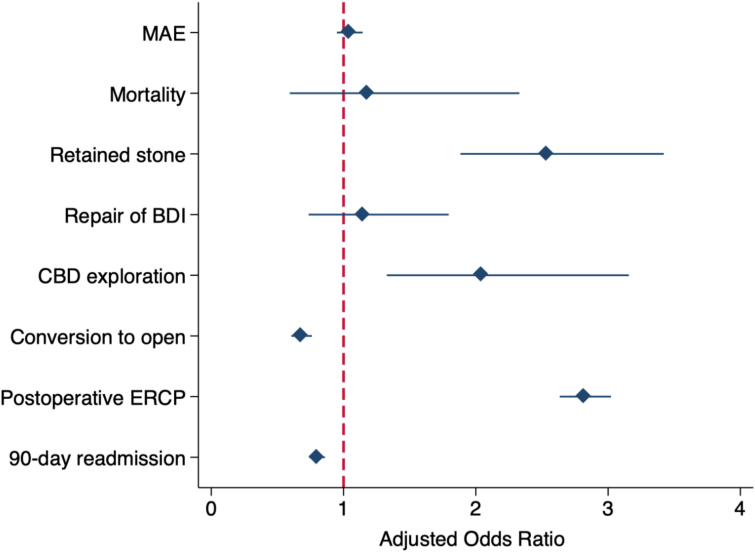
Risk-adjusted outcomes associated with use of intraoperative cholangiography (IOC) during cholecystectomy for mild gallstone pancreatitis. *MAE: Major adverse events are a composite of mortality, any complications, and repair of bile duct injury (BDI). CBD: Common bile duct. ERCP: Endoscopic Retrograde Cholangiopancreatography.*

Following risk adjustment for the covariates shown in Supplemental Table 3, the use of IOC was associated with over 2-fold increased odds of retained stone (AOR 2.54 [95 % CI 1.88–3.42]), common bile duct exploration (2.05 [1.33–3.16]), and postoperative ERCP (2.82 [2.63–3.02], Fig. 2). Of note, the odds of MAE, mortality, complications, and repair of bile duct injury remained comparable between *IOC* and *No IOC* (Supplemental Table 4). In addition, IOC was significantly associated with over 30 % decreased odds of conversion to open (AOR 0.68 [95 % CI 0.61–0.76]). On adjusted analysis of financial outcomes, LOS, hospitalization costs, and non-home discharge were comparable between the two groups (Supplemental Table 4).

### Readmission outcomes

**Fig. 3:**
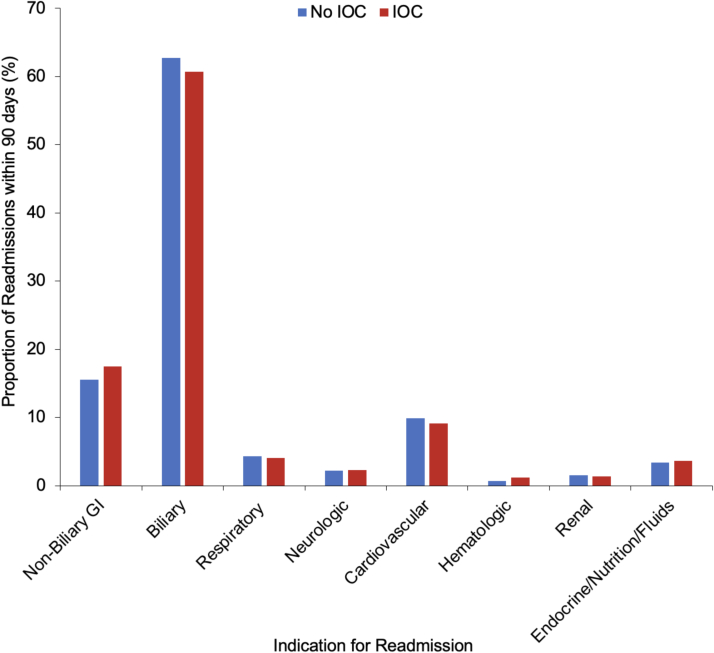
Indications for readmission within 90 days of discharge following cholecystectomy for mild gallstone pancreatitis, stratified by the use of intraoperative cholangiography (IOC). *GI: Gastrointestinal.*

Compared to those without IOC, patients with IOC experienced significantly lower rates of 90-day readmission (7.2 vs 8.9 %, *p* < 0.001, Table 2). Approximately 60 % of readmissions were due to recurrent pancreatitis, retained stone, ERCP, biliary sepsis, or other hepatobiliary indication (Fig. 3). Non-biliary gastrointestinal complications comprised approximately 15 % of readmissions. Of note, indications for readmission remained similar regardless of the use of IOC (*p* = 0.57, Fig. 3). Furthermore, total costs including index hospitalization and readmission were comparable between the two cohorts (*IOC:* $14,900 [IQR: 11,200-20,900] vs *No IOC:* $14,900 [IQR: 11,100-21,000], *p* = 0.05).

**Fig. 4:**
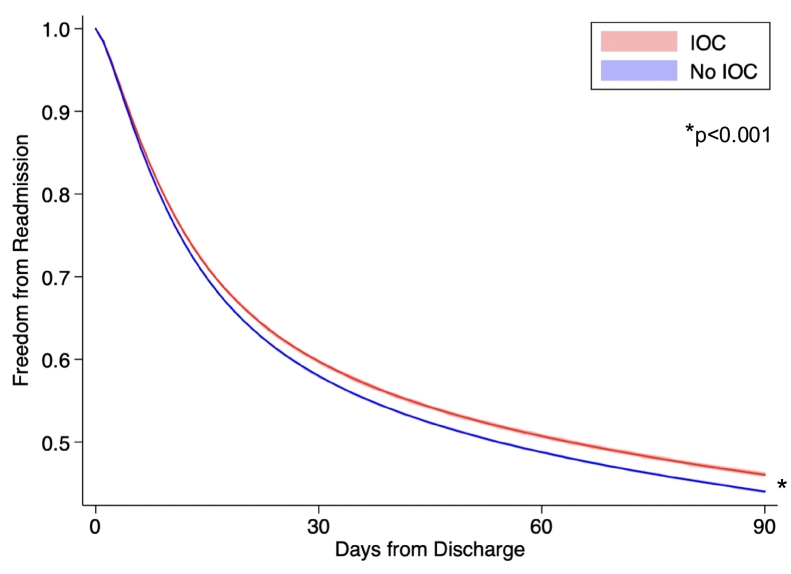
Royston-Parmar time- and risk-adjusted hazard analysis for 90-day nonelective readmission following cholecystectomy for mild gallstone pancreatitis, stratified by use of intraoperative cholangiography (IOC).

**Fig. 5:**
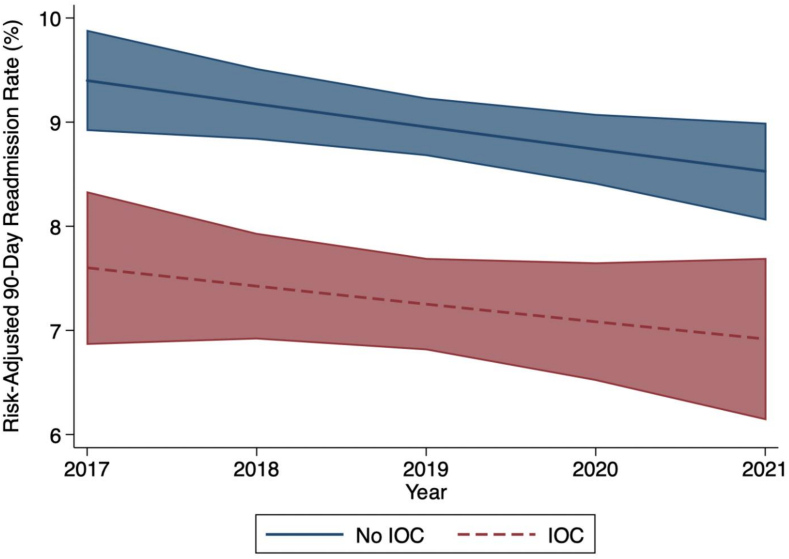
Temporal trend in risk-adjusted rate of 90-day readmission following cholecystectomy for mild gallstone pancreatitis, stratified by use of intraoperative cholangiography (IOC).

Following risk adjustment*, IOC* was associated with approximately 20 % reduced odds of 90-day readmission relative to *No IOC* (AOR 0.80 [95 % CI 0.74–0.86], Fig. 2, Supplemental Table 4). These findings were confirmed on Royston-Parmar analysis, which demonstrated significantly decreased hazard of readmission within 90 days of discharge with the use of IOC (*p* < 0.001, Fig. 4). While overall rates of 90-day readmission decreased over time, the disparity in readmission between the *IOC* and *No IOC* cohorts persisted over the study period (Fig. 5).

## Discussion

The role of common bile duct evaluation with IOC in patients with mild gallstone pancreatitis remains controversial. Using a nationally representative cohort of patients undergoing cholecystectomy for mild GSP, the present study characterized the utilization of IOC and its association with in-hospital clinical and financial outcomes. Over the 5-year study period, the utilization of IOC significantly decreased from 26.5 % in 2017 to 20.7 % in 2021. IOC was more frequently performed at high-volume, private centers. Notably, rates of major adverse events, including mortality, complications, and repair of bile duct injury, in addition to LOS were comparable between patients with and without IOC. In addition, IOC was significantly associated with 20 % reduced odds of 90-day readmission, which was predominantly for recurrent biliary disease. Furthermore, total costs including index hospitalization and readmission were comparable. Several of these findings merit further discussion.

A major consequence of omitting IOC during cholecystectomy for GSP is the failure to detect concomitant choledocholithiasis and the potential risk of readmission for recurrent pancreatitis or biliary obstruction. This risk is highlighted in the present study by the 20 % lower odds of 90-day readmission associated with use of IOC. Common bile duct stones have previously been found in as many as 13–16 % of patients with mild to moderate GSP who underwent IOC [[22], [23], [24]]. In the absence of cholangitis or obstructive jaundice, the clinical significance of these CBD stones remains debated. Gallstones that cause GSP tend to be smaller than those causing cholangitis, so some surgeons may argue these smaller stones will pass into the bowel without need for intervention. Several prior smaller studies have demonstrated no difference in the rate of recurrent pancreatitis or biliary complications between GSP patients who had IOC and those who did not [8,10,13]. However, a systematic review of RCTs found that routine IOC had superior perioperative CBD stone detection and significantly reduced readmission for retained CBD stones [9]. Of note, the study was not limited to patients with GSP. Similarly, we noted 9 % of patients without IOC requiring rehospitalization within 90 days of discharge, compared to 7 % of patients with IOC. Given the sparse readmission rates of 0–1 % in a prior 2016 multi-center study of IOC for GSP, our national analysis may provide a more accurate representation of variation in practice patterns and may better capture readmissions [10]. Our findings suggest that IOC significantly reduces risk for readmission and should be incorporated into the management of patients with mild GSP.

Notably, patients with and without IOC demonstrated comparable mortality, complications, LOS, and overall hospitalization costs, suggesting that IOC use may ultimately be a cost-effective treatment approach. One concern causing hesitancy to perform IOC is the prolonged operative time and additional associated costs [25]. However, the present national study demonstrated a modest $100 increase in index hospitalization costs including IOC and subsequent procedures (CBD exploration and postoperative ERCP) and no impact on LOS as compared to no IOC. Our findings are consistent with previous cost estimates for performing IOC ranging from $100–700 [[25], [26], [27]]. After accounting for the significant reduction in readmissions, total expenditures including the cost of readmission were comparable between cohorts. A 2011 national cost-effectiveness analysis comparing the management of CBD stones found cholecystectomy with IOC to be the cost-effective treatment strategy if the probability of CBD stones was >4 % [28]. Given the etiology of GSP and high likelihood for retained choledocholithiasis, IOC may be particularly beneficial for this patient population undergoing cholecystectomy. In the setting of mild GSP, IOC appears to be favorable with no impact on complications, hospital stay, or costs and a significant reduction in gallstone-related readmissions.

Another concern some surgeons may raise is the increased surgical risk potentially associated with IOC and subsequent procedures to remove CBD stones. Both IOC and CBD exploration require advanced operative skills to be performed laparoscopically [29]. Positive findings on IOC may prompt the need for CBD exploration, which may contribute to greater rates of conversion to open as demonstrated in the present study [30]. Nevertheless, conversion to open rates were highest among patients without IOC, suggesting that the benefits in clarifying biliary anatomy with IOC may outweigh the risk of prompting additional procedural interventions. Moreover, the 30 % reduced odds of conversion to open associated with IOC further contributes to cost savings by avoiding the complications associated with an open operation [31]. As conversion to open CBD exploration has been shown to correlate with surgeon experience, greater utilization of IOC across the nation may help increase familiarity with the technology, minimize surgical risks, and improve outcomes [32].

Declining utilization of IOC in recent years may reflect the rise of alternative modalities to assess for choledocholithiasis. Trending laboratory values, such as liver function tests and bilirubin levels, particularly on hospital day 2 may help indicate persistent CBD stones [7,33]. The use of preoperative ERCP in the absence of cholangitis or obstructive jaundice is debated, and IOC has been shown to have shorter hospital stay, lower costs, and similar treatment failure rate [34]. Furthermore, magnetic resonance cholangiopancreatography (MRCP) is a noninvasive strategy to detect retained CBD stones, which may preclude the need for IOC with normal imaging findings [7,35]. However, obtaining additional imaging and preoperative ERCP if needed can delay surgery, and a 2022 randomized trial found that IOC was associated with shorter hospital stay, higher stone detection rate, and no difference in costs or complications compared to MRCP [35]. Alternatively, positive or equivocal MRCP may also trigger the use of IOC and have contributed to the observed rates of stone detection. Of note, ICG angiography, used in lieu of IOC to enhance visualization of biliary anatomy, was shown to increase in use over the study period and may partially explain the decline in IOC use [36]. Further comparative effectiveness studies to evaluate different modalities of cholangiography not captured in this dataset, such as MRCP and intraoperative ultrasound, may help improve outcomes for patients with GSP [37].

The decreasing trend in readmission among all patients may reflect better overall selection for IOC in the contemporary era. The use of noninvasive alternative modalities to identify choledocholithiasis, such as laboratory value trends, preoperative MRCP, or intraoperative ultrasound, are likely contributing to the observed improvement in readmission even without IOC [7,[33], [34], [35], [36], [37]]. Nevertheless, we still observed persistently lower readmission rates among patients with IOC, highlighting the beneficial role of this modality in identifying retained CBD stones. While the rates of retained stones and CBD exploration were quite low (0.1–0.5 %), the rate of postoperative ERCP among patients with IOC (11.4 %) was most similar to prior literature estimates of 13–16 % and appeared to best capture the presence of choledocholithiasis [[22], [23], [24]]. The lower rate of postoperative ERCP observed in the current study was consistent with the overall decreasing trend in readmission, further suggesting improvements in the methods of identifying patients at risk of retained CBD stones in recent years.

Enhancing access to IOC and incorporating routine use into surgical management for mild GSP may improve detection of clinically relevant CBD stones. We found that IOC was associated with treatment at high-volume, private hospitals, highlighting a disparity in utilization. This finding is consistent with prior literature demonstrating significant variation in IOC use attributable to the surgeon and hospital, rather than patient characteristics [25]. Surgeon decision-making may be influenced by skill and preference, including the ability to perform laparoscopic IOC and CBD exploration and familiarity with biliary anatomy [38]. Moreover, hospital factors such as availability of ERCP, radiology providers, and fluoroscopic technology play a significant role in the use of IOC [39]. Thus, hesitance to perform IOC may be greatest at low-volume centers where fluoroscopy is not readily available, contributing to delays in care and greater financial implications [40]. Standardized guidelines recommending routine IOC may serve as a systems-level intervention to detect suspected or unsuspected CBD stones in mild GSP, prevent development of biliary complications, and improve value of care. Further efforts to increase availability of local resources to perform IOC particularly at low-volume, public centers are warranted.

This study has several limitations due to its retrospective nature and use of an administrative database. The NRD lacks clinical granularity regarding laboratory values such as preoperative liver function tests, bilirubin levels and imaging findings regarding CBD size, which would have better characterized the severity of acute pancreatitis and presence of persistent choledocholithiasis. Factors such as operative time, reason for conversion to open, radiology availability, and surgeon experience were unable to be ascertained. The use of alternative modalities such as preoperative magnetic resonance cholangiopancreatography (MRCP) and intraoperative ultrasound were also not captured. Our analysis was limited to the duration of each admission and did not include outpatient data. Additionally, ICD coding may be influenced by variation in provider and hospital practices. For example, ICD codes for retained stone and CBD exploration were particularly sparse and may have underestimated their incidence. Nevertheless, we analyzed multiple data elements including postoperative ERCP to more accurately estimate the presence of choledocholithiasis. Furthermore, history of ERCP on prior admission was unable to be assessed. Despite these limitations, we used the largest, all-payer readmissions database and robust statistical methods to enhance the generalizability of our findings at the national level.

In conclusion, the present study used a nationally representative database to demonstrate that IOC use during cholecystectomy for mild GSP was associated with 20 % reduced odds of 90-day readmission for complications related to a retained stone. Notably, patients with and without IOC experienced comparable complications, LOS, and hospitalization costs. Our findings suggest that IOC significantly decreases risk of recurrent biliary disease following mild GSP and is cost-effective. Given the decreasing utilization of IOC over time, increasing access to fluoroscopic technology particularly among low-volume hospitals may help improve outcomes. Further studies are needed to determine whether routine use of IOC should be incorporated into standardized guidelines for management of mild GSP.

## CRediT authorship contribution statement

**Ayesha P. Ng:** Writing – review & editing, Writing – original draft, Visualization, Validation, Methodology, Investigation, Formal analysis, Data curation, Conceptualization. **Troy N. Coaston:** Writing – review & editing, Methodology, Formal analysis, Conceptualization. **Konmal Ali:** Writing – review & editing, Writing – original draft, Formal analysis. **Christian de Virgilio:** Writing – review & editing, Supervision, Investigation, Conceptualization. **Peyman Benharash:** Writing – review & editing, Supervision, Software, Resources, Project administration, Methodology, Conceptualization.

## Ethics approval

This study was deemed exempt from full review by the Institutional Review Board at the University of California, Los Angeles.

## Funding sources

The present work did not receive any funding.

## Declaration of competing interest

The authors declare no conflicts of interest.

## Appendix A Supplementary data

Supplementary tables Image 1
