# Supplementary material for: National trends in utilization and readmission following intraoperative cholangiography in gallstone pancreatitis
Source: Surg Open Sci. 2025 May 10;26:79–86. doi: 10.1016/j.sopen.2025.05.002 (PMC12143823; doi:10.1016/j.sopen.2025.05.002)
Supplement: Supplementary file 1 — Supplementary tables [file mmc1.docx]

Supplemental Table 1. Administrative *International Classification of Diseases, 10^th^ Revision* (ICD-10) diagnosis and procedure codes for cholecystectomy for mild gallstone pancreatitis.

|  | **ICD-10** |
| --- | --- |
| **Mild gallstone pancreatitis** | K85.90, K85.10, K85.80, K85.00 |
| **Cholecystectomy** |  |
| Laparoscopic | 0FT44ZZ, 0FB44ZZ, 0FB48ZZ, 0F544ZZ, 0F548ZZ |
| **Intraoperative cholangiography** | BF030ZZ, BF031ZZ, BF03YZZ, BF0C0CZZ, BF0C1ZZ, BF0CYZZ, BF100ZZ, BF10YZZ, BF110ZZ, BF111ZZ, BF11YZZ, BF120ZZ, BF121ZZ, BF12YZZ, BF130ZZ, BF131ZZ, BF13YZZ, BF140ZZ, BF141ZZ, BF14YZZ, BF180ZZ, BF181ZZ, BF18YZZ |
| **Exclusions** |  |
| Cholangitis | K80.3x, K83.0x |
| Hepatic, biliary, pancreatic, and duodenal malignancy | C22, C23, C24, C25 |
| Liver transplant (procedure) | 0FY00Z0, 0FY00Z1, 0FY00Z2 |
| Liver transplant (diagnosis) | T86.4 |
| ERCP | 0FJB8ZZ, 0FJD8ZZ, 0FB58ZX, 0FB68ZX, 0FB78ZX, 0FB88ZX, 0FB98ZX, 0FBC8ZZ, 0F758ZZ, 0F768ZZ, 0F778ZZ, 0F788ZZ, 0F798ZZ, 0F7C8ZZ, 0F9C8ZZ, 0F758DZ, 0F768DZ, 0F778DZ, 0F788DZ, 0F798DZ, 0F7C8DZ, 0F9580Z, 0F9680Z, 0F9780Z, 0F9880Z, 0F9980Z, 0F9C80Z, 0FC58ZZ, 0FC68ZZ, 0FC78ZZ, 0FC88ZZ, 0FC98ZZ, 0FCC8ZZ, 0FF48ZZ, 0FF58ZZ, 0FF68ZZ, 0FF78ZZ, 0FF88ZZ, 0FF98ZZ, 0FFC8ZZ, 0FCD8ZZ, 0FCF8ZZ, 0FFD8ZZ, 0FFF8ZZ, 0F9D80Z, OF9F80Z |
| **End-organ dysfunction** |  |
| Hypotension | I95 |
| Acute respiratory failure | J96 |
| Altered mental status | R40 |
| Renal failure | N17, N19 |
| Acute hepatic dysfunction | K72.0 |
| Thrombocytopenia | D96.5, D69.6 |
| **Comorbidities** |  |
| Diabetes | E10, E11, E13 |
| Hypertension | I10, I11, I12, I13, I15 |
| Coronary artery disease | I20, I24, I25 |
| Congestive heart failure | I43, I50, I09.9, I11.0, I13.0, I25.5, I42.0, I42.5, I42.6, I42.7, I42.8, I42.9, P29.0 |
| Chronic lung disease | I27.8, I27.9, J68.4, J70.1, J70.3, J40, J41, J42, J43, J44, J45, J46, J47, J60, J61, J62, J63, J64, J65, J66, J67 |
| Chronic kidney disease | Z49, N19, I12.0, N25.0, Z94.0, N18.5, N18.6, I13.11, V45.1, Z99.2, Z91.15 |
| **Treatment Characteristics** |  |
| Common bile duct exploration | 0FC90ZZ, 0FC50ZZ, 0FC60ZZ, 0FC70ZZ, 0FC80ZZ, 0FF53ZZ, 0FF57ZZ, 0FF60ZZ, 0FF63ZZ, 0FF67ZZ, 0FF70ZZ, 0FF73ZZ, 0FF77ZZ, 0FF80ZZ, 0FF83ZZ, 0FF87ZZ, 0FF90ZZ, 0FF93ZZ, 0FF97ZZ, 0FFC0ZZ, 0FFC3ZZ, 0FFC7ZZ |
| Indocyanine green (ICG) angiography | 4A1BXSH, BF50x, BF52x, BF53x, BF5Cx |
| Conversion to open | Z53.31, 0F540ZZ, 0F543ZZ, 0FB40ZZ, 0FB43ZZ, 0FT40ZZ |
| Repair of bile duct injury | 0F15-9, 0FQ5-9, 0FR5-9, 0FU5-9 |
|  |  |
| **Complications** |  |
| Retained stone | K91.86 |
| Sepsis | A40, A41, R65.20, T814XXA, K68.11 |
| Abscess | R78.81, K63.0 |
| Wound infection | T81.32XA, T81.31XA, T81.4XXA, K68.11 |
| Pneumonia | J12, J13, J14, J15, J16, J18, J95.851, J95.89 |
| Pneumothorax | J95.811 |
| Acute respiratory distress syndrome | J80, R06.03 |
| Respiratory Failure | J96.00, J96.90, J96.20, J95.821, J95.822 |
| Prolonged Mechanical Ventilation | 5A1955Z |
| Deep Vein Thrombosis | I82.220, I82.4, I82.6, I82.A1, I82.B1, I82.C1, I82.290, I82.890, I82.91, I80.9, I80.3 |
| Pulmonary Embolism | I26 |
| Cardiac arrest | I46.2, I46.8, I46.9 |
| Ventricular tachycardia | I47.2 |
| Ventricular fibrillation | I49.01 |
| Cardiac tamponade | I31.4 |
| Myocardial infarction | I21 |
| Hemorrhage | D78.01, D78.02, D78.21, D78.22, E36.01, E36.02, E89.810, E89.811, G97.31, G97.32, G97.51, G97.52, H59.111, H59.112, H59.113, H59.119, H59.121, H59.122, H59.123, H59.129, H59.311, H59.312, H59.313, H59.319, H59.321, H59.322, H59.323, H59.329, H95.21, H95.22, H95.41, H95.42, I97.410, I97.411, I97.418, I97.42, I97.610, I97.611, I97.618, I96.620, J95.61, J95.62, J95.830, J95.831, K91.61, K91.62, K91.840, K91.841, L76.01, L76.02, L76.21, L76.22, M96.810, M96.811, M96.830, M96.831, N99.61, N99.62, N99.820, N99.821 |

Supplemental Table 2. Diagnosis-related group (DRG) codes used to define indications for readmission following cholecystectomy for mild gallstone pancreatitis.

|  | **DRG** |
| --- | --- |
| **Biliary indications** |  |
| Biliary sepsis / infectious complications | 870, 871, 872, 862, 863, 853, 856, 857, 858, 602, 603, 689, 690, 695, 696, 853, 854, 855, 867, 868, 869, 977 |
| Retained stone | 444, 445, 446 |
| ERCP | 408, 409, 410 |
| Gallstone pancreatitis | 438, 439, 440 |
| Other hepatobiliary diagnosis/procedure | 441, 442, 443, 432, 433, 434, 438, 439, 440, 417, 418, 419, 411, 412, 413, 414, 415, 416, 417, 405, 406, 407, 423, 424, 425, 420 |
| **Non-biliary GI indications** |  |
| Gastrointestinal bleed | 377, 378, 379 |
| Bowel obstruction | 388, 389, 390 |
| Esophagitis or gastroenteritis | 392, 391 |
| Stomach or bowel procedure | 326, 327, 328, 329, 330, 331, 344, 345, 346, 394, 393, 395, 371, 372, 373, 335-337, 374-376, 356-358, 381-387, 347-358, 338-349, 368-370, 380-387 |
|  |  |
| Respiratory | 193, 194, 195, 177, 178, 179, 186, 187, 188, 189, 180, 181, 182, 208, 207, 204, 205, 206, 190, 191, 192, 202, 203, 166, 167, 168, 004, 199, 200, 201 |
| Neurologic | 025, 026, 027, 069, 067, 068, 070, 071, 072, 061, 062, 063, 064, 065, 066, 056, 057, 091, 092, 093, 074, 073, 100, 101, 102, 103, 040, 041, 042, 037, 038, 039, 021, 020, 022, 149, 055, 054 |
| Cardiovascular | 292, 291, 293, 280, 281, 282, 283, 284, 285, 311, 313, 312, 308, 309, 310, 247, 246, 248, 249, 250, 251, 175, 176, 286, 287, 314, 315, 316, 299, 300, 301, 283, 284, 285, 294, 295, 002, 216, 217, 218, 219, 220, 221, 233, 234, 235, 236, 242, 243, 244, 245, 163, 164, 165, 303, 302, 228, 229, 230, 215, 003 |
| Hematologic | 808-816, 820-830, 834-836 |
| Renal | 685, 698, 699, 700, 727, 728, 725, 726, 682, 683, 684, 686, 687, 688, 674, 673, 675, 671, 672, 665, 666, 667, 668, 669, 670, 656, 657, 658, 659, 660, 661 |
| Endocrine, nutrition, or fluid disorder | 640, 641, 643, 644, 645, 637, 638, 639, 642 |

Supplemental Table 3. Patient and hospital characteristics associated with use of intraoperative cholangiography (IOC) during cholecystectomy for mild gallstone pancreatitis. *Ref: Reference. AOR: Adjusted odds ratio. CI: Confidence interval.*

| **Parameter** | **AOR [95% CI]** | ***p*-value** |
| --- | --- | --- |
| Age (per year) | 1.00 [0.99-1.00] | 0.42 |
| Female sex | 1.02 [0.99-1.06] | 0.19 |
| *Income Quartile* |  |  |
| Fourth (highest) | Ref |  |
| Third | 1.05 [0.99-1.12] | 0.13 |
| Second | 1.07 [1.00-1.15] | 0.05 |
| First (lowest) | 1.02 [0.95-1.10] | 0.61 |
| *Payer Status* |  |  |
| Private | Ref |  |
| Medicare | 0.96 [0.90-1.01] | 0.10 |
| Medicaid | 0.83 [0.78-0.88] | <0.001 |
| Uninsured | 1.00 [0.92-1.08] | 0.97 |
| Other | 1.07 [0.97-1.19] | 0.18 |
| *Comorbidities* |  |  |
| Elixhauser Comorbidity Index | 0.99 [0.99-1.01] | 0.49 |
| Diabetes | 0.95 [0.91-1.00] | 0.07 |
| Hypertension | 0.99 [0.92-1.07] | 0.80 |
| Coronary artery disease | 1.09 [1.02-1.16] | 0.01 |
| Chronic lung disease | 1.04 [0.98-1.10] | 0.16 |
| Chronic kidney disease | 0.93 [0.77-1.11] | 0.41 |
| Year of admission | 0.94 [0.91-0.96] | <0.001 |
| *Hospital Volume Status* |  |  |
| Low | Ref |  |
| Medium | 1.09 [1.01-1.17] | 0.02 |
| High | 1.15 [1.05-1.26] | 0.003 |
| *Hospital Teaching Status* |  |  |
| Non-metropolitan | Ref |  |
| Metropolitan non-teaching | 0.76 [0.68-0.85] | <0.001 |
| Metropolitan teaching | 0.73 [0.66-0.82] | <0.001 |
| *Hospital Ownership* |  |  |
| Government | Ref |  |
| Private non-profit | 1.09 [0.98-1.22] | 0.11 |
| Private investor-owned | 1.45 [1.27-1.65] | <0.001 |

Supplemental Table 4. Risk-adjusted outcomes associated with use of intraoperative cholangiography (IOC) among patients undergoing cholecystectomy for mild gallstone pancreatitis. *AOR: Adjusted odds ratio. ß: Beta coefficient. CI: Confidence interval. LOS: Length of stay. ERCP: Endoscopic Retrograde Cholangiopancreatography. MAE: Major adverse events are a composite of mortality, any complications, and repair of bile duct injury. ICG: Indocyanine green.*

| **Outcome** | **AOR or ß [95% CI]** | **p-value** |
| --- | --- | --- |
| MAE | 1.04 [0.95-1.14] | 0.38 |
| In-hospital mortality | 1.18 [0.59-2.33] | 0.64 |
| *Complications* |  |  |
| Retained stone | 2.54 [1.88-3.42] | <0.001 |
| Infectious | 1.07 [0.86-1.32] | 0.55 |
| Respiratory | 0.90 [0.78-1.05] | 0.17 |
| Cardiac | 1.02 [0.78-1.34] | 0.90 |
| Hemorrhagic | 0.97 [0.77-1.21] | 0.76 |
| Thromboembolic | 0.82 [0.58-1.16] | 0.26 |
| Repair of bile duct injury | 1.15 [0.74-1.80] | 0.54 |
| Common bile duct exploration | 2.05 [1.33-3.16] | 0.001 |
| Conversion to open | 0.68 [0.61-0.76] | <0.001 |
| Postoperative ERCP | 2.82 [2.63-3.02] | <0.001 |
| LOS (days) | -0.1 [-0.1, -0.02] | 0.01 |
| Costs ($) | +200 [-10-400] | 0.06 |
| Non-home discharge | 0.98 [0.89-1.09] | 0.77 |
| 90-day readmission | 0.80 [0.74-0.86] | <0.001 |
